# Exercise intolerance and fatigue in chronic heart failure: is there a role for group III/IV afferent feedback?

**DOI:** 10.1177/2047487320906919

**Published:** 2020-02-11

**Authors:** Luca Angius, Antonio Crisafulli

**Affiliations:** 1 Faculty of Health and Life Sciences, Sport, Exercise and Rehabilitation, Northumbria University, UK; 2 Department of Medical Sciences and Public Health, Sports Physiology Laboratory, University of Cagliari, Italy

**Keywords:** Metabo-reflex, fatiguability, circulation, exercise pressor reflex, sensory neurons, muscle fatigue

## Abstract

Exercise intolerance and early fatiguability are hallmark symptoms of chronic heart failure. While the malfunction of the heart is certainly the leading cause of chronic heart failure, the patho-physiological mechanisms of exercise intolerance in these patients are more complex, multifactorial and only partially understood. Some evidence points towards a potential role of an exaggerated afferent feedback from group III/IV muscle afferents in the genesis of these symptoms. Overactivity of feedback from these muscle afferents may cause exercise intolerance with a double action: by inducing cardiovascular dysregulation, by reducing motor output and by facilitating the development of central and peripheral fatigue during exercise. Importantly, physical inactivity appears to affect the progression of the syndrome negatively, while physical training can partially counteract this condition. In the present review, the role played by group III/IV afferent feedback in cardiovascular regulation during exercise and exercise-induced muscle fatigue of healthy people and their potential role in inducing exercise intolerance in chronic heart failure patients will be summarised.

## Introduction

Exercise intolerance and early fatiguability are hallmark symptoms of chronic heart failure (CHF). These symptoms severely limit daily activities and have been traditionally considered as the consequence of the inability of the heart to meet the metabolic demand of the muscles during exercise, with the malfunction of the heart as a pump as the leading cause.^1^ However, the pathophysiological mechanisms of exercise intolerance in CHF are more complex, multifactorial and only partially investigated and understood.

At the heart level, the combination of systolic and diastolic abnormalities concurs in reducing the capacity to increase cardiac output (CO). Moreover, several other abnormalities such as impairments in peripheral endothelial-dependent vasodilation, reduction in systemic oxygen delivery, reduction in pulmonary reserve and respiratory muscle perfusion have all been observed and they potentially account for the reduced exercise capacity in this syndrome.^2–5^

Changes in skeletal muscle metabolism, functioning, composition and architecture have also been described and, in recent years, there has been mounting evidence that these changes play a pivotal role in the development of exercise intolerance and in the reduction of exercise capacity. Some clues point towards the existence of a peripheral reflex that becomes hyperactive secondary to the described skeletal muscle alterations, and may contribute to the exercise intolerance and the early fatiguability experienced by these patients.^5,6^ In particular, it has been reported that some hormonal systems (i.e. renin–angiotensin–aldosterone, vasopressin and atrial natriuretic peptide) are overactivated and that patients show altered autonomic nervous system activity at rest and during exercise, with exaggerated sympathetic tone associated with parasympathetic withdrawal. While the exact mechanisms causing this hormonal and autonomic dysregulation are still to be fully elucidated, it appears that an exaggerated afferent feedback from group III/IV muscle afferents may be at least in part responsible for this dysregulation.^5–8^ Importantly, physical inactivity appears to affect the progression of the syndrome negatively, while physical training can partially reverse this condition. Enhancements in exercise capacity observed after physical training appear to be the consequence of improvements in muscle and vascular function rather than cardiac function. Although effective in reducing mortality, classic pharmacological treatments, such as angiotensin-converting enzyme inhibitors, β-blockers and diuretics show very limited or null effects on exercise capacity.^3,6,9–12^

To date, most of the literature investigating the role of group III/IV muscle afferents has focused on the physiology and pathophysiology of cardiovascular regulation during exercise. Only recently a series of experiments has emphasised their role on muscle fatigue development and in the inhibition of central motor drive.^13,14^ Thus, overactivity of feedback from group III/IV muscle afferents may cause exercise intolerance with a double action: by inducing cardiovascular dysregulation and by facilitating the development of muscle fatigue. In the present narrative review, we will summarise the role of group III/IV afferent feedback in cardiovascular regulation during exercise and exercise-induced muscle fatigue of healthy people, and their potential role in inducing exercise intolerance in CHF patients.

## Role of group III and IV muscle afferents in cardiovascular regulation during dynamic exercise in healthy individuals

In healthy individuals, the cardiovascular adjustment to dynamic exercise is characterised by an increase in heart rate (HR) and stroke volume (SV), which together enhance CO. At the same time, a profound reduction in systemic vascular resistance (SVR) takes place due to metabolite-induced vasodilation in the working muscle. As result, mean arterial pressure (MAP) remains stable or slightly increases.^15–17^ Behind these haemodynamic changes there is a fine tuning operated by neural mechanisms.

In particular, at least three neural mechanisms concur in this physiological response. One is a central mechanism, commonly known as ‘central command’. In this mechanism, the cardiovascular control areas located in the brainstem are reflexively activated by regions of the brain responsible for motor unit recruitment. Central command is believed to establish a basal level of sympathetic activity and parasympathetic withdrawal to the cardiovascular apparatus closely linked to the exercise intensity.^18,19^

This basic pattern of autonomic activity is in turn modulated by a second mechanism arising from peripheral signals originating from type III/IV muscle afferents in the muscle, which act as mechano and metaboreceptors. Group III/IV nerve endings represent more than 50% of the total muscle afferents and constitute the sensory arm of a reflex which is collectively termed the ‘exercise pressor reflex’ (EPR). These muscle afferents convey information about the mechanical and metabolic variations of the contracting muscle via the spinal cord to the cardiovascular control centres within the brainstem.^18,20–22^ It was reported that most group III afferents act mainly as ‘mechanoreceptors’ as they respond to mechanical distortion, whereas group IV afferents appear to respond to metabolite accumulation, so that they can be considered as ‘metaboreceptors’ as well as nociceptors. Several substances such as lactic acid, potassium, bradykinin, arachidonic acid products, ATP, diprotonated phosphate and adenosine are thought to stimulate the metaboreceptors in the muscle.^23,24^ It should be noted that a subpopulation of group III/IV nerve endings respond to both mechanical and chemical stimuli.^25,26^ Experimental evidences suggest that mechanoreceptors can be sensitised by metabolite accumulation making it difficult to isolate their pure mechano from metabo properties.^23,27^ Group III/IV muscle afferents project to the dorsal horn of the spinal cord. However, little is known about their projections at the cortical and subcortical level but it seems that the medulla oblongata is essential for its function.^22,28,29^

The activation of both central command and EPR leads to autonomic adjustments characterised by an increase in sympathetic activity and parasympathetic withdrawal. This autonomic regulation is in turn modulated by the third reflex operating during exercise: the baroreflex. Arterial baroreceptors are located in the carotid sinus bifurcation and aortic arch and sense rapid changes in blood pressure thereby activating the baroreflex. When arterial blood pressure is acutely increased or reduced, the baroreceptors are stretched or compressed, and this deformation causes increment or reduction in the afferent neuronal firing rate, respectively. The control over blood pressure is achieved by reflexively inducing rapid adjustments in HR and SVR in response to changes in MAP.^30,31^ The baroreflex activity avoids any excessive variation in blood pressure and opposes any mismatch between vascular resistance and CO.^32,33^

One interesting point of the functioning of these reflexes is their interaction during exercise, as both the central command and the EPR can modulate the activity of the baroreflex.^34^ In detail, during exercise the operating point of the baroreflex is shifted and that the stimulus response curve is relocated to a higher arterial blood pressure in direct relation to exercise intensity, without any change in its sensitivity.^30,35,36^ In short, the baroreflex is still operating during exercise, but the blood pressure operating point is higher than rest, although it is as effective as at rest in controlling blood pressure.^30,34,37^

It is remarkable that the target blood pressure can often be achieved despite a lack in response of one of the regulated variables, thereby suggesting that these reflexes operate with a high level of effectiveness and integration.^18,38,39^ For example, it has been demonstrated in human investigations dealing with EPR that when cardiac contractility cannot be enhanced, the possibility to increase SV and CO is precluded. Then, the target blood pressure is achieved by recruiting the SVR reserve (i.e. by inducing arteriolar vasoconstriction). Similarly, if venous return is impaired and/or the reserve in cardiac preload is exploited, then the recruitment of the SVR reserve is the main mechanism through which the EPR operates to adjust haemodynamics.^40^ In short, it appears that whenever SV and CO can not properly increase (such as in CHF patients), then exaggerated arteriolar constriction becomes the main mechanism through which the target blood pressure is reached. In contrast, in healthy individuals the preferred cardiovascular adjustment during EPR is a flow-mediated (i.e. CO-mediated) mechanism obtained by recruiting the inotropic and preload reserves.^40^

Hence, during exercise, central command, EPR and baroreflex are all activated and complex interaction occurs between these reflexes. While it is well ascertained that some redundancy and neural occlusion exist between EPR and central command (i.e. their effects do not sum), it is also remarkable that they can modulate the activity of the other two. As previously shown, the most studied interaction is the modulation of baroreflex operated by central command and EPR. However, interaction has also been demonstrated between central command and EPR. Some evidence suggests that inputs from type III/IV muscle afferents modulate the central command activity and exert an inhibitory effect on central motor drive.^41^ In particular, it was observed that a reduction in afferent input from type III/IV muscle afferents during exercise obtained with epidural anaesthesia resulted in an increase in central command activity. These findings support the concept that central command cannot work properly without adequate feedback from peripheral muscles and that, at the same time, this feedback limits central command and motor drive.^13,41^

## Role of group III and IV muscle afferents in the development of muscle fatigue during dynamic exercise in healthy individuals

Sustained physical exercise inexorably leads to a reduced capacity to generate maximal force or power. This has been commonly described as muscle fatigue.^14^ Most muscle fatigue has been documented to occur at or distal to the neuromuscular junction,^42^ and has been commonly defined as peripheral fatigue. Conversely, central fatigue refers to the inability of the central nervous system to optimally recruit the muscle (e.g. a significant decrease in voluntary activation).^14^ Supraspinal fatigue, a subset of central fatigue, can be described as a suboptimal output from the motor cortex.^14^ Consequently, the interaction between central and peripheral fatigue leads to a decrease in maximal force or power. The role and interplay of central and peripheral fatigue in exercise intolerance has been the object of discussion for several years.^43,44^ Peripheral fatigue is measured by comparing the force of the muscle elicited by electrical stimulation of the corresponding motor nerve before and after exercise.^45^ Quantification of central fatigue is generally performed with the superimposed twitch technique.^46^ More recently, transcranial magnetic stimulation (TMS) has been effectively used to study and quantify supraspinal fatigue.^47^

Several experiments have been performed to understand and identify the physiological mechanisms contributing to the generation of muscle fatigue.^14,43^ A considerable amount of experimental evidence supports the hypothesis that during high intensity exercise, the activity of group III/IV muscle afferents might facilitate the development of central fatigue via an inhibitory feedback at different sites of the motor pathway and also by influencing the level of motor unit activation.^41,48^ Injection of hypertonic saline in the muscle has been classically used as an experimental approach to stimulate type III/IV muscle afferents. A reduction of the low-threshold motor unit discharge rate during low intensity muscular contraction^49,50^ and maximal force production of knee extensor^50^ and elbow flexors muscles^51^ has been found. Other studies have reported a decrease in motor evoked potentials elicited by TMS, thus providing evidence of an inhibitory effect at the supraspinal level.^52^ The reduction in maximal force production seems to be caused by a decrease in voluntary activation (e.g. increase in central fatigue). This hypothesis seems to be confirmed in studies in which voluntary activation was significantly reduced in elbow flexors muscles.^51^ On the other hand, other studies have reported an increase in spinal motoneuron excitability.^53^ Overall, these studies have shown that motor unit activation can be partially regulated by peripheral reflexes elicited by group III/IV muscle afferents.

Post-exercise circulatory occlusion has also been employed as an alternative experimental approach to stimulate group III/IV muscle afferents. In this regard, early investigations have demonstrated that maximal voluntary contraction and voluntary activation were significantly reduced and did not recover until the circulation was restored.^54–57^ Interestingly, when the fatigued muscle was kept ischaemic, the decline in maximal force and voluntary activation was also present in unfatigued muscle of the same limb.^55,56^ These findings suggest the presence of a convergence and divergence effect at the spinal level,^58^ where muscle afferents from one muscle may have projections to the dorsal horn receiving inputs from other adjacent muscles. Studies involving TMS have demonstrated that the decline in maximal force production and voluntary activation was also caused by an inhibitory effect at the supraspinal level.^55,56,59^

Pharmacological blockade has also been adopted as an experimental approach to study the role of group III/IV muscle afferents.^41^ This intervention performed prior to whole-body exercise, is able to attenuate approximately 60% of feedback from these afferents. Overall, these investigations have reported that during high intensity cycling exercise, voluntary drive (estimated by means of electromyography), metabolite accumulation and peripheral fatigue increased when the feedback from exercising muscles was attenuated.^60–62^ It should be noted that in these studies physical performance was unchanged or impaired when afferent feedback was attenuated, and this was probably the consequence of an impaired cardiorespiratory response.^41,61,63^ More recently, a series of experiments involving TMS reported an increase in corticospinal excitability when feedback from exercising muscles was attenuated.^64–66^ These preliminary findings seem to confirm previous experiments showing that group III/IV muscle afferents promote central fatigue during exercise.

## Group III and IV muscle afferents and their role in cardiovascular regulation and fatiguability in patients with CHF

During EPR, several abnormalities in the cardiovascular regulation have been demonstrated in individuals with CHF. To study the metaboreflex, i.e. the metabolic part of the EPR, some human studies have employed the post-exercise muscle ischaemia method, which induces metabolite accumulation thereby stimulating type III/IV muscle afferents in the muscle. During metaboreflex activation in patients with CHF, an increase in MAP similar to that observed in healthy individuals was found. However, the mechanisms underlying this cardiovascular response were markedly different between patients and controls. In detail, in patients with CHF the rise in MAP was reached by a SVR increase, while in healthy individuals this was the result of a flow increment, i.e. CO elevation.^67^ This observation was recently also reported in patients with heart failure with preserved ejection fraction^68^ as well as in patients with coronary artery disease.^69^ This abnormal haemodynamics seemed the consequence of the incapacity of CHF patients to recruit the reserves in cardiac performance and in cardiac pre-load in response to the metaboreflex. The exaggerated increase in SVR (i.e. arteriolar constriction) compensated for the inability to increase SV. Interestingly, in CHF patients the MAP response was well preserved notwithstanding their lower CO in comparison with healthy individuals. Hence, it appears that CHF causes a functional shift from flow-mediated (i.e. CO increase) to vasoconstriction-mediated (i.e. SVR increase) in the mechanisms by which the target blood pressure is reached during EPR. It is to be noted that this haemodynamic scenario closely resembles what has also been reported in animal models of CHF.^70–72^

It is to be emphasised that the described exaggerated arteriolar constriction in response to metaboreflex potentially restrains muscle perfusion,^73^ and this probably contributes to the early development of fatigue and exercise intolerance shown by CHF patients. In detail, while in healthy individuals metaboreflex activation maintains skeletal muscle perfusion,^73^ this is not the case in CHF, in which exaggerated vasoconstriction takes place during the metaboreflex. In this scenario, even the exercising muscle may become vasoconstricted,^67,74,75^ and this occurrence may lead to deleterious consequences in terms of exercise tolerance and muscle tropism.

The so-called ‘muscle hypothesis’ has been proposed to explain at least in part the exercise intolerance shown by CHF patients. In detail, it has been suggested that CHF initiates a vicious circle in which damage to the heart and disturbance in central haemodynamics trigger compensatory mechanisms, including neurohumoral and sympathetic activation, which persistently vasoconstricts the muscle circulation. In the longer term, this condition becomes harmful and the damage at the vascular and endothelial level develops, with chronic inflammation and necrosis at muscular level. Various signs of myopathy, muscle mass reduction and abnormal metabolic and mechanical functions are actually present in CHF.^76,77^ Importantly, these muscle abnormalities correlate better with exercise tolerance compared to measures of left ventricular function.^74^ The described muscle abnormalities in turn cause elevation in the feedback from III/IV afferents during muscle contraction, and this heightens EPR activity and dysregulates haemodynamics, with excessive arteriolar constriction and muscle hypoperfusion. In short, in CHF central haemodynamic abnormalities in response to EPR initiate a vicious circle which, in the longer term, causes muscle hypoperfusion, muscle wasting and a reduction in strength.^5,78^

In support of the ‘muscle hypothesis’ there are experimental findings in heart transplant recipients during metaboreflex activation. In these patients exercise capacity, although improved, remains abnormally impaired after transplant compared to normal individuals. This indicates that restoring cardiac function does not fully enhance aerobic metabolism. A possible explanation for the incomplete recovery might be the muscular abnormalities which developed before transplant and still persist after transplant, thereby impairing exercise capacity in these patients.^79,80^ Moreover, it has been observed that an improvement in exercise capacity after heart transplant was paralleled by improvements in cardiovascular response to metaboreflex, with a gradual reduction in the metaboreflex-induced arteriolar constriction.^79^ Collectively, these findings in heart transplant recipients seem to indicate that the EPR is dysregulated in these patients before transplant and that this dysregulation tends to ameliorate several months after transplant, in parallel with muscle metabolism and functions. This observation appears to be in line with the ‘muscle hypothesis’ of CHF.

Some authors have argued against the concept that metaboreflex is accentuated in the CHF syndrome. In particular, they have proposed that mechanoreceptor rather than metaboreceptor stimulation is responsible for the abnormal haemodynamics observed in CHF during EPR activation.^81–83^ It has also been proposed that metaboreflex control of sympathetic activity is attenuated in CHF and that metaboreceptors are desensitised in this syndrome.^28^ It is likely that the conflicting results in the scientific literature on whether muscle metaboreflex is attenuated or accentuated may depend on the degree of muscle abnormalities of the CHF population, the degree of metaboreceptor desensitisation and the mode of exercise being performed. Furthermore, authors reporting attenuated metaboreflex and accentuated mechanoreflex employed an animal model of CHF (mainly rats), thus the application of these findings in humans is contentious. Moreover, evidence suggests that mechanoreceptors are sensitised by metabolites, thus rendering it difficult to differentiate the role of mechanoreflex from that of metaboreflex.^27^ Finally, it should be noted that, in the human model, most studies dealing with metaboreflex employed the post-exercise muscle ischaemia method to assess metaboreflex function. This manoeuvre probably rules out any contribution of mechanoreceptors because they do not operate in this setting.

Whether the mechanoreflex or the metaboreflex is responsible for the abnormal elevation in EPR activity, the EPR is dysfunctional in these patients. It is interesting to note that Amann and colleagues^84^ were able to demonstrate that lumbar intrathecal fentanyl reduced the excessive vascular resistance during knee extensor exercise in a CHF population, thereby demonstrating a role of type III/IV muscle afferents in the abnormal haemodynamics in this syndrome. More recently, van Iterson and colleagues^85^ reported that blocking type III/IV muscle afferents with intrathecal fentanyl in CHF patients resulted in a faster oxygen consumption kinetics. They hypothesised that the slower oxygen consumption kinetics of CHF was the consequence of peripheral and central haemodynamic maldistribution due to abnormal group III/IV muscle afferent activation and that blocking these afferents could at least partially restore a normal exercise response.

Concerning the role played by type III/IV muscle afferents on exercise capacity, it should be emphasised that very few studies have been conducted in a clinical population.^84,86^ Gagnon and colleagues^86^ were the first to investigate the exercise response following their pharmacological blockade in chronic obstructive pulmonary disease. Contrary to what was found in a healthy population, endurance time during a cycling constant work rate was enhanced by an average of 215 seconds. According to the authors, the reduced and delayed hyperventilatory response and lowered perceived dyspnoea during exercise might provide a possible explanation for the increased exercise capacity. Furthermore, a deeper loss in quadriceps muscle strength and a higher lactate level after exercise with spinal anaesthesia was also found. In CHF patients, Amann and colleagues^84^ found an increase in vascular conductance, peripheral blood flow (15% increase), and leg oxygen delivery together with a significant reduction in MAP following intrathecal fentanyl. Interestingly, the decline in maximal force production of the quadriceps following exercise was attenuated by 30% compared to the control condition. Taken together, these findings suggest that in these kinds of clinical population, type III/IV afferent feedback might play a pivotal role to reduce exercise tolerance.^6,87,88^

One phenomenon recently proposed to explain the potential influence of group III/IV afferents on muscle fatigue development is their ability to affect cerebral blood flow and oxygenation. It has been proposed that EPR activation lowered cerebral perfusion by counteracting the normal vasodilation occurring at the brain level during exercise. This would increase the sense of effort and impair motor drive.^9^ However, the regulation of cerebral circulation is complex, and more research is warranted to understand better the phenomenon and to confirm this hypothesis.

Experimental studies have proposed that afferent feedback from ventilatory muscles might be important for exercise intolerance in CHF patients. Both animal and human models have reported histological and biochemical alterations of the diaphragm muscle in CHF.^89^ Respiratory muscle dysfunction is typically observed in CHF patients and this is often called respiratory muscle weakness, which is caused by a substantial reduction in respiratory muscle strength (in particular the diaphragm).^90^ During exercise, CHF patients develop early diaphragmatic fatigue therefore limiting the ventilatory response and so reducing pulmonary gas exchange and oxygen delivery. Furthermore, increased breathlessness, exertional dyspnoea from low exercise intensities are typically observed. Similar to the locomotor muscles, an exaggerated metaboreflex from the espiratory muscle has been observed.^91^ This mechanism, also described as inspiratory muscle metaboreflex, is particularly important during sustained exercise as it modulates the competition for blood flow between the locomotor and respiratory muscles.^92^ This mismatch potentially leads to an increase in ventilatory work and may exacerbate exertional dyspnoea and exercise intolerance. Taken together, these respiratory muscle abnormalities have been shown to contribute to the early development of fatigue in CHF patients.^90^

**Figure 1.**
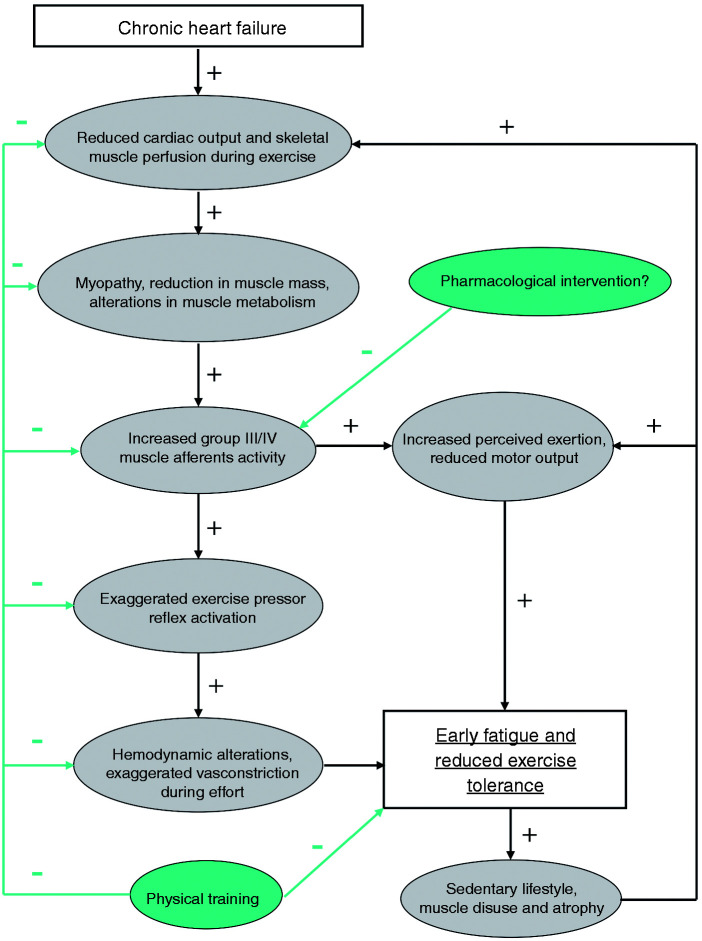
Putative mechanisms responsible for the exercise intolerance and early fatigue induced by type III/IV afferent feedback in chronic heart failure. Abnormal central haemodynamics initiate a cascade which ultimately results in an increase in III/IV afferent feedback activity. It is to be emphasised that physical training may potentially counteract this malfunctioning at various levels, while, to date, no pharmacological intervention has been demonstrated to be able to correct this abnormal regulation.

It should be noted that the number of studies is very limited and therefore the precise role of group III/IV muscle afferents contributing to the development of muscle fatigue and exercise tolerance in the clinical population is largely unknown. Nevertheless, these preliminary studies provide encouraging evidence that these muscle afferents can be the target of future therapeutic strategies. The major putative mechanisms responsible for the exercise intolerance and early fatigue induced by type III/IV afferent feedback in CHF are shown in Figure 1.

## Exercise training as therapy for CHF patients

Aerobic exercise training is recognised as an important adjunct for improving the quality of life of CHF patients. The benefits of aerobic training have been discussed in previous reviews.^93,94^ Aerobic training has been demonstrated to affect positively maximal oxygen consumption, central and peripheral haemodynamic function, peripheral vascular function and muscular function. These adaptations result in a higher workload for the same HR and perceived exertion.^93^ Continuous aerobic training of 45–60 minutes’ duration is well tolerated and recommended for CHF patients. Interval/intermittent aerobic training has recently been shown to be more effective. This involves less than 5 minute bouts at 90–95% of the maximal exercise capacity interspaced by more than 3 minutes of recovery.^94^

Resistance training has also been introduced to counteract the decline in functional alteration and muscle mass of the locomotor muscles. Previous works recommended an intensity of 30–40% of one repetition max (1RM) and a rating of perceived exertion (RPE) less than 12 for increasing local aerobic capacity, while 40–60% of 1RM and RPE less than 15 for increasing muscle mass. It is important to state that aerobic exercise remains the main training and therefore resistance training can be considered as a complement.^94^

As respiratory muscle weakness is inversely correlated with exercise capacity, interventions with the potential to improve respiratory muscle strength might be able to counter this condition. Recent studies have focused on the role of respiratory muscle training for the improvement of respiratory muscle strength in CHF. Several beneficial effects such as an improvement in maximal inspiratory capacity, improvement in peripheral and respiratory oxygen supply and a reduction in exertional dyspnoea have been reported together with improved exercise capacity and exercise tolerance with a better quality of life.^98^ Winkelmann et al.^99^ have shown that inspiratory muscle training combined with aerobic training was more beneficial than aerobic training alone in CHF patients. However, the optimal training regimen is yet to be defined. However, it seems that the benefits of respiratory muscle training appear to be intensity-dependent suggesting that high intensity training regimens are required to obtain an improvement in aerobic capacity.^100^ Interestingly, a study performed by Chiappa and colleagues^91^ showed that inspiratory muscle training was capable of decreasing limb vasculature resistance by increasing the blood flow of limb muscles at rest and during exercise. One of the main results of these adaptations was the attenuation of metaboreflex activity.^91^ Other mechanisms might also be responsible for the increase in exercise capacity in CHF patients such as resting left ventricular function, endothelial vasodilator function and improved ventilatory response. However, other mechanisms are yet to be elucidated.

In animal models of CHF (rats), it was demonstrated that exercise exerts beneficial effects on the exaggerated EPR, although the underlying mechanisms have not been completely elucidated.^95,96^ Observations in humans have reported that exercise training could reverse the exaggerated exercise-induced sympathetic activity, vasoconstriction and ventilatory drive in patients with CHF.^97^ More recently, these observations have been confirmed and support the concept that exaggerated EPR activity is at least in part responsible for the sympathetic overactivity in CHF patients, and that this condition can be successfully counteracted by exercise training.^95^ However, it is to be acknowledged that the mechanisms of exercise-mediated beneficial effects on EPR remain largely unknown in humans. Further studies are warranted to prove definitively whether exercise training is effective in reducing the exaggerated EPR in these patients.

## Conclusions and future directions

In summary, the pathophysiological mechanisms of exercise intolerance and exercise-induced muscle fatigue in patients with CHF are complex and involve peripheral and central factors. In this context, reflexes mediated by group III/IV muscle afferents appear to play an important role in the phenomenon. The exaggerated afferent feedback coming from these fibres potentially causes haemodynamic dysregulation, with excessive sympatho-excitation and arteriolar constriction. The abnormally elevated neural feedback may also exacerbate the rate of development of peripheral and central fatigue by causing a restriction in motoneuronal output. Whether this exaggerated feedback arises from the activity of mechano or metaboreceptors is still a matter of debate. There is also the possibility that both receptors are involved in the phenomenon. Future study specifically designed is necessary to unravel this question.

Although demonstrated in a few studies, exercise-induced muscle fatigue in the clinical population can be partially attributed to a higher and/or abnormal activity of group III/IV muscle afferents and can also explain the reduced exercise capacity and exercise intolerance of patients with CHF. In light of these findings, further studies are required to elucidate the mechanisms of group III/IV muscle afferents at various sites of the motor pathway and peripheral level during exercise.

It is important that future investigations take into account the possible effects of pharmacological therapy on the correction of type III/IV muscle afferent hyperactivity. From a clinical perspective it would be useful to verify whether blockade of these receptors limits the excessive sympathetic excitation and the reduced motor output observed in CHF patients. To the best of our knowledge, a drug that specifically blocks these afferents has yet to be tested in humans. Another further field for future research is the effect of physical training on type III/IV afferent activity. Such an investigation would reveal whether a physical training programme would dampen the hyperactivity shown by these muscle afferents in CHF syndrome. This would have the practical consequence to test whether the prescription of physical activity and the adoption of an active lifestyle is an effective means to treat exercise intolerance and to reduce fatiguability in these patients.
